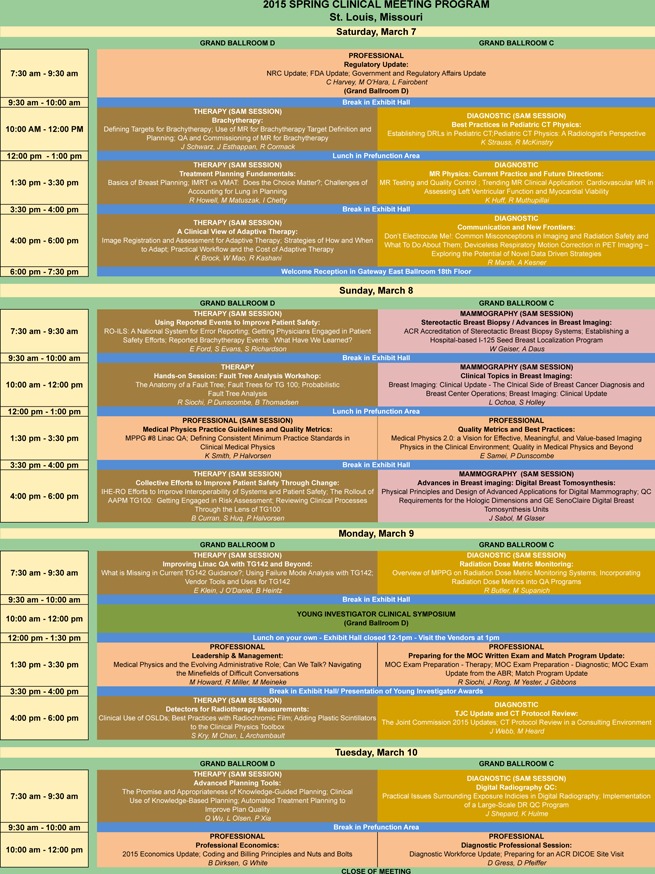# MEETING PROGRAM

**DOI:** 10.1120/jacmp.v16i3.5829

**Published:** 2015-05-15

***Available on‐line at*** www.aapm.org/meetings/2015SCM/

**2015 AAPM Spring Clinical Meeting March 7 – 10, 2015**

**St. Louis, MO**

**Chair**

Brian Wang, PhD

University Louisville Louisville, KY

**Vice Chair**

Jessica B. Clements, MS

Kaiser Permanente Los Angeles, CA

**Track Directors**

**Therapy Track**

Jean M. Moran, PhD

University Michigan Medical Center Ann Arbor, MI

Kyle J. Antes, MS

Presbyterian Healthcare System Dallas, TX

**Professional Track**

Jessica B. Clements, MS

Kaiser Permanente Los Angeles, CA

Michael Howard, PhD

Sarah Cannon Cancer Center Chattanooga, TN

**Diagnostic Track**

Dustin Gress, MS

MD Anderson Cancer Center Houston, TX

Jeffrey M. Moirano, MS

University of Washington Seattle, WA

**Young Investigator Program**

Jean M. Moran, PhD

University Michigan Medical Center Ann Arbor, MI

Brian Wang, PhD

University Louisville Louisville, KY

David E. Hintenlang, PhD

University of Florida Gainesville, FL

**Mammography Track**

Jessica B. Clements, MS

Kaiser Permanente Los Angeles, CA

Dustin Gress, MS

MD Anderson Cancer Center Houston, TX

**Figure 1:**